# 

**Published:** 1893-12-23

**Authors:** 


### CHRISTMAS APPEAL NUMBER.

### CONTENTS.

**England and Her Christmas** - 177
**Around the Hospitals** - 178
**On Modern Progress in Ophthalmic Medicine and Surgery.** By Robert Brudenell Carter, F.R.C.S. - 179
**Christmas Cheer** - 180
**Figures that Feel and Work** - 181
**Annotations.**—The Air of Schools—Life on Threepence Three-Farthings a Day—Birds and Big Dogs - 183
**The Hospital Clinic—**
Nature and Treatment of Flat Foot - 185
**Notes and News** - 184
**Sweet Charity's Guide for Christmas Givers** - 186
General Hospitals.—
Special Hospitals.—Consumption—Epilepsy and Paralysis—Children—Lying-In—Women—Miscellaneous—Special - 189
A Few Other Charities - 191

### EXTRA SUPPLEMENT.—THE NURSING MIRROR.

**News from the Nursing World.**—Our Princess—Christmas Greetings — Christmas Festivities — Precarious Pleasures — Queen's Nurses in Scotland—A Needful Addition—Incurables Well Cared For - cxi

**Christmas Books** - cxii

**Help the Nurses to Help the Sick** - cxiii

**Christmas Day.** The District Nurse - cxiv

**Christmas Day with a Private Case** - cxv

**Appointments** - cxv

**Christmas at Biarritz** - cxvi

**Christmas in a Workhouse Infirmary** - cxvi

**Nellie's Christmas** - cxvii

**Christmas Day in Japan** - cxvii

**Christmas Carol** - cxviii

**How Bobbie Spent Christmas** - cxviii

**The Book World for Women and Nurses** - cxix

